# Aroma improvement by repeated freeze-thaw treatment during *Tuber melanosporum* fermentation

**DOI:** 10.1038/srep17120

**Published:** 2015-11-26

**Authors:** Deng-Rong Xiao, Rui-Sang Liu, Long He, Hong-Mei Li, Ya-Ling Tang, Xin-Hua Liang, Tao Chen, Ya-Jie Tang

**Affiliations:** 1 Key Laboratory of Fermentation Engineering (Ministry of Education), Hubei Provincial Key Laboratory of Industrial Microbiology, Hubei University of Technology, Wuhan 430068 China; 2 State Key Laboratory of Oral Diseases, West China Hospital of Stomatology, Sichuan University, Chengdu 610041 China; 3 Key Laboratory of Systems Bioengineering (Ministry of Education), School of Chemical Engineering and Technology, Tianjin University, Tianjin 300072 China

## Abstract

The aroma attributes of sulfurous, mushroom and earthy are the most important characteristics of the aroma of *Tuber melanosporum*. However, these three aroma attributes are absent in the *T. melanosporum* fermentation system. To improve the quality of the aroma, repeated freeze-thaw treatment (RFTT) was adopted to affect the interplay of volatile organic compounds (VOCs). Using RFTT, not only was the score on the hedonic scale of the aroma increased from the “liked slightly” to the “liked moderately” grade, but the aroma attributes of sulfurous, mushroom and earthy could also be smelled in the *T. melanosporum* fermentation system for the first time. A total of 29 VOCs were identified, and 9 compounds were identified as the key discriminative volatiles affected by RFTT. Amino acid analysis revealed that methionine, valine, serine, phenylalanine, isoleucine and threonine were the key substrates associated with the biosynthesis of the 9 key discriminative VOCs. This study noted that amino acid metabolism played an important role in the regulation of the aroma of the *T. melanosporum* fermentation system.

*Tuber melanosporum*, known as “black diamond,” is highly appreciated in many cuisines due to its combination of smooth texture, pungent odor/perfume, and musty earthy flavor^1^,^2^,^3^,^4^. Sulfurous and pungent notes, contributed by dimethyl sulfide and 2-methylbutanal, are of great importance to the final aroma impression^5^,^6^,^7^. And mushroom note, contributed by 1-Octen-3-ol, is also known for the characteristic black Périgord truffle aroma^8^,^9^. As reported by Culleré *et al.*^10^, seven representative aroma attributes were selected to describe the black Périgord truffle aroma, namely sulfur, mushroom, mold, animal, boiled potatoes, butter and cheese. The sensual, seductive and unique aroma consists of a series of volatile organic compounds (VOCs). They are not synthesized merely for the pleasure of humans but also act as odorant cues for other mammals and insects to locate the precious fungi underground and spread their spores^6^,^8^. More than 200 VOCs have been described in various truffle species. These VOCs are hydrocarbons with a high vapor pressure, generally including alcohol, aldehyde and/or ketone functional groups, and often containing sulfur atoms^5^,^6^,^9^,^10^,^11^,^12^,^13^.

Due to their distinctive characteristics and high economic value, each kilogram of truffles sells on the open market for €600–€6000, depending on the species^9^. However, truffle cultivation is notoriously difficult, in part because of its cryptic life cycle as an underground symbiont^4^. Over the years, many attempts have been made, and our lab has established a submerged fermentation technique for *Tuber* production^14^,^15^,^16^,^17^,^18^,^19^. For the *T. melanosporum* fermentation system, a total of 59 VOCs were identified, including 53 from the broth and 32 from the fresh mycelia. The VOCs included alcohols, aldehydes, ketones, esters and acids, aromatic compounds, sulfur, nitrogen, isoprenoids, ethers, furanes and indenes. Compared to the VOCs of the truffle fruiting body, alcohol-derived compounds were predominant in both the fermentation mycelia and the broth. In the mycelia, long-chain fatty acids and isoprenoids were primarily found, while in the broth, sulfur compounds, pyrazines, furans and jasmones contributed to the intense wine bouquet properties^20^. Through sensory evaluation, five aroma attributes were determined to describe the fermentation aroma, namely ethanol, flowery, fruity, green and sweet. This result indicated that some differences in the VOCs and aroma attributes remained between *T. melanosporum* fermentation and the fruiting body, and further research was needed to improve the quality of the fermentation aroma.

The concentration variation of metabolic substances of VOCs or the overexpression of related genes can impact the formation of VOCs, resulting in differences in the aroma^21^,^22^. By adding 5 g/L L-methionine to the fermentation medium, six volatile organic sulfur-containing compounds (methanethiol, dimethyl sulfide, dimethyl disulfide, dimethyl trisulfide, 3-(methylthio)propanal and 3-(methylthio)-1-propanol) with great importance in the final aroma impression were first detected in the submerged fermentation of *T. melanosporum*^22^. However, previous research found that intact *Aspergillus flavus* conidia scarcely synthesized C8 volatiles, but repeated freeze-thaw treatment (RFTT) that rendered the cell membrane permeable promoted (R)-1-octen-3-ol formation, which was responsible for the mushrooms of *T. melanosporum*. It was speculated that the enzymes were not active in intact fungal tissues or cells or lacked access to the substrates (linoleic acid and oxygen), perhaps due to the localization of the enzymes and substrates in different intracellular compartments^23^. RFTT was employed for cell disruption. Repeated temperature fluctuations can cause a number of degradative effects. Even minor temperature changes can cause water to form ice crystals, which then melt and recrystallize, damaging the integrity of the cellular compartments. Thus, the cellular membranes lose their osmotic status and their semi-permeability. During this process, a variety of damaging mechanisms can occur, including dehydration, oxidation, syneresis, off-flavor development and textural breakdown, depending on the extent of freeze–thaw cycling and the type of food in question^23^,^24^,^25^,^26^,^27^.

To improve the quality of the fermentation aroma, RFTT was adopted to treat the fermentation sample. First, a sensory evaluation was conducted to judge the effects of RFTT on the aroma attributes of fermentation. Then, the VOC profile was dynamically analyzed to identify the components of the aroma. Immediately afterward, principal component analysis (PCA) and univariate analysis were conducted to identify the key compounds that induced the improvement in the aroma by RFTT. Finally, potential substrate metabolism (amino acid metabolism) and VOC production were analyzed based on the Kyoto Encyclopedia of Genes and Genomes (KEGG) and research studies to reveal the important roles of key VOC metabolisms in the formation of aroma.

## Results and Discussion

**Figure 1:**
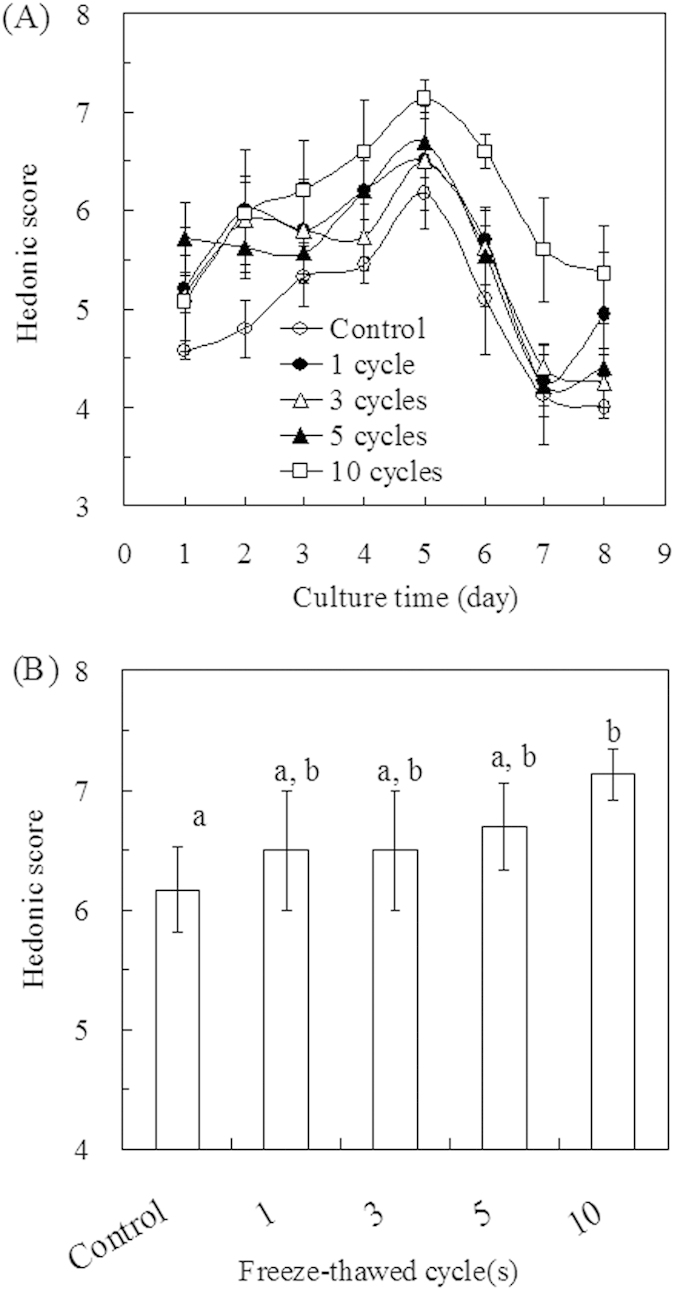
Effects of freeze-thaw cycle(s) on *T. melanosporum* fermentation aroma. (**A**) Hedonic scores of fermentation samples with different freeze-thaw cycles during the fermentation process, and (**B**) comparison of highest values of each freeze-thaw cycle in (**A**). The Scheffe multiple-range tests (α ≤ 0.05) were employed to assess whether significant differences existed between the individual variables, and different letters (e.g., a, b) and their combinations (ab) in (**B**) were assigned to significantly different groups.

A general assessment of fermentation samples using different freeze-thaw conditions was performed. To investigate the effects of RFTT on the fermentation aroma, a sensory evaluation involving hedonic scores and aroma attributes was conducted by four expert panelists to provide a global view of the aroma variability. As shown in Fig. 1A, the hedonic scores of groups of samples all showed the same tendency—a gradual increase from Days 1 to 5, with the acquisition of the highest value on Day 5, and an immediate drop at the end of fermentation. It was clear that the hedonic scores of the fermentation sample with RFTT were higher than the control, especially for fermentation with 10 freeze-thaw cycles. This result indicated that RFTT might have a positive effect on the aroma quality. All groups of fermentation samples reached their highest values on Day 5. Compared to the 6.17 score of the control, hedonic scores showed a significant improvement, with 7.13 for the fermentation sample with 10 freeze-thaw cycles (Fig. 1B). The aroma quality was most favorable on Day 5, and the aroma of fermentation samples frozen at −20 °C for 55 min and then thawed at 30 °C for 5 min over 10 cycles was significantly improved.

To clarify the characteristics of the fermentation aroma, aroma attributes were also studied. The original fermentation aroma mainly presented five aroma attributes, namely ethanol, flowery, fruity, green and sweet (Table 1). For the fermentation sample treated with one freeze-thaw cycle, a sulfurous odor was detected on Days 3 and 6. With increasing freeze-thaw cycles, earthy, sulfurous and mushroom odors could be discriminated from the original five aroma attributes on the first four cultivation days. With ten freeze-thaw cycles, a mushroom aroma existed in the fermentation aroma from Days 1 to 3, and a sulfurous odor could be detected on Days 3 and 4. At the end stage of fermentation, the earthy aroma could be discriminated from other aroma attributes.

Based on sensory evaluation, RFTT had a definite positive effect on the aroma of fermentation. The hedonic acceptability was increased from the “liked slightly” (6.17) to the “liked moderately” (7.13) grade, accompanied by the enrichment of the fermentation aroma with sulfurous, earthy and mushroom odors.

Upon tracing the material basis of the aroma, a total of 29 VOCs were identified (without chirality determination) during the *T. melanosporum* fermentation process: 26 from the fermentation samples without RFTT (control) and 24 from the fermentation samples with RFTT. Table 2 shows the identified compounds and their relative intensities. These compounds included alcohols, aldehydes, ketones, esters and acids, aromatic compounds, nitrogen, sulfur and alkene. During the fermentation process, aromatic compounds, alcohols and nitrogen were the predominant VOCs (Supplementary Fig. S2). The aromatic compounds were identified as the major constituents in this work, corresponding to more than 23% of the total VOCs. The total relative intensity decreased from Days 2 to 5 and then increased on Day 7 by RFTT. Among the 7 aromatic compounds identified, 2-phenyl-ethanol was the primary component. This compound was also the key component of the *T. melanosporum* fruiting body and mainly presents the smell of rose flowers^13^. Aromatic compounds were derived from the catabolism of phenylalanine via the Ehrlich pathway^6^.

Seven alcohol compounds were identified, namely ethanol, 2-methyl-1-propanol, 3-methyl-1-butanol, 4-hexen-1-ol, 1-octen-3-ol, 3-octanol and [S-(Z)]-3, 7, 11-trimethyl-1, 6, 10-dodecatrien-3-ol. Alcohols were the predominant group, comprising no less than 22% of the total VOCs. When treated with RFTT, the alcohols’ total relative content increased from Days 2 to 7. The alcohols mainly arose from amino acid metabolism and lipid oxidation processes^6^,^8^.

The nitrogen series, providing a welcome roasted flavor, is always present in fermented soybeans, cocoa, and cheese^28^,^29^,^30^. Five compounds were identified in this study, and the total relative content changed little with RFTT during the fermentation process. Categorically, there are two widely accepted mechanisms for the formation of pyrazines: the Strecker degradation of α-amino acids and reductones and the ammonia/acyloin reaction of ammonia and α-hydroxycarbonyls^31^.

**Figure 2:**
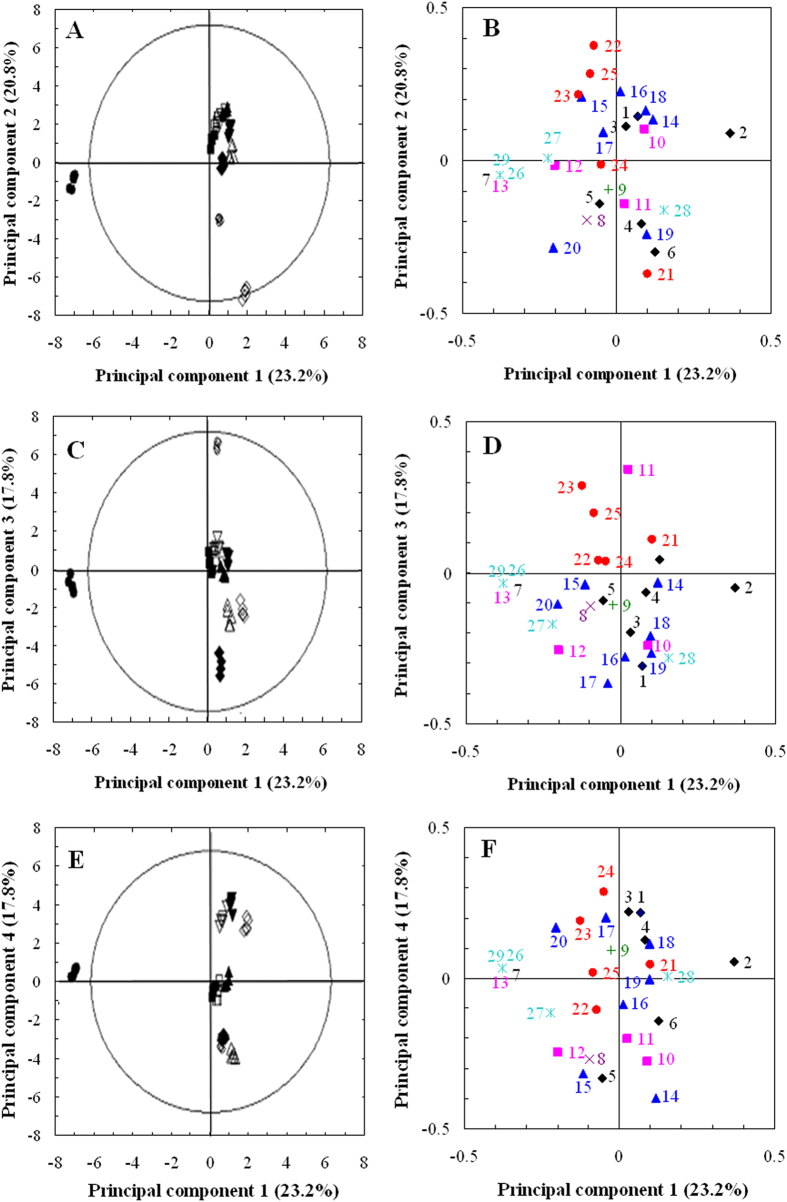
PCA of VOCs of *T. melanosporum* fermentation samples. Five fermentation time-points were selected and treated with the RFTT. Score and loading plots for the first, second, third and fourth principal components are shown. PC1 (23.2%) was plotted against PC 2 (20.8%) (**A**,** B**), against PC3 (17.8%) (**C**,** D**), and against PC4 (13.7%) (**E**, **F**) to produce the PC score plots and loading plots. Symbols for different treatment fermentation samples: Day 2-control (closed circle, ●), Day 2-RFTT (open circle, ○), Day 3-control (closed square, ■), Day 3-RFTT (open square, □), Day 4-control (closed triangle, ▲), Day 4-RFTT (open triangle, △), Day 5-control (closed diamond, ♦), Day 5-RFTT (open diamond, ⋄), Day 7-control (closed inverted triangle, ▼), Day 7-RFTT (open inverted triangle,▽).

A multivariate statistical method, PCA, was applied to the VOC dataset for a graphical summary of the changes in abundance of VOCs between the different fermentation samples and to assess the technical, analytical, and biological reproducibility. The PCA score plot indicated that the levels of technical, analytical, and biological reproducibility were extremely high, as assessed by the close superimposition of the technical and biological replicates (Fig. 2A, C, E). The PCA score plot (PC1 × PC2) clearly revealed the closer relationships of Days 3, 4, 5 and 7 compared to Day 2. The aroma on Day 2 was distant from the others, where the main aroma descriptor was medium. The samples from Days 3, 4, 5 and 7 were dispersed from each other on PC3 and PC4, indicating differences in the VOCs of these fermentation samples. When focusing on the effect of RFTT on the aroma, it was clear that the samples with RFTT were distant from the samples without RFTT on PC1, PC2, PC3 and PC4. This result reflected differences in the VOCs of the fermentation samples with RFTT. The interpretation of the PCA loading plot (Fig. 2B, D, F) allowed mining of the VOC variables that contributed most to the separation of the fermentation samples observed in the PCA score plot. Significant VOC differences among fermentation samples became even clearer within these location-based models. The loading values of the four principal components are shown in Supplementary Table S2 and indicated that 28 out of the 29 detected VOCs were discriminative compounds.

In addition to multivariate analyses performed using PCA, a univariate analysis (Kruskal–Wallis test) was performed to test for significant VOC differences among the ten samples. VOCs that were deemed discriminant within the PCA loading plots and that were also significantly different at the 95% significance level according to the Kruskal–Wallis test were deemed significant VOCs warranting further investigation. Following this data-mining regime, 28 of the 29 detected VOCs were considered significant.

There was a genotypic difference in metabolic profiles between the control and RFTT samples at each selected time-point (Days 2, 3, 4, 5 and 7). Based on the results of the PCA and the Kruskal–Wallis test, 17 discriminative compounds were identified from the sample on Day 2 with RFTT, 9 from the sample on Day 3 with RFTT, 9 from the sample on Day 4 with RFTT, 13 from the sample on Day 5 with RFTT, and 5 from the sample on Day 7 with RFTT. Following the comparison and mining of these discriminative compounds, 9 (Nos. 5, 6, 12, 14, 15, 16, 24, 25 and 27) of the 29 VOCs were selected as the most discriminative VOCs, which included alcohols, esters, aromatic compounds, nitrogen compounds and alkenes (Supplementary Table S3).

The biosynthetic pathways of a great number of volatile constituents could be traced back to the primary metabolism, with carbohydrates, fatty acids, and especially amino acids representing the natural carbon pools for the fermentation aroma. It is known that a great range of alcohols, aldehydes, aromatic compounds and nitrogen compounds are derived from the degradation of aromatic and branched-chain amino acids^32^,^33^. Branched-chain amino acids such as leucine are known precursors for aldehydes such as 3-methyl-butanal, whereas aromatic amino acids such as phenylalanine coulde produce alcohol and aldehyde volatile constituents through decarboxylation reactions^32^. For example, phenylalanine acted as the metabolic precursor of ethylbenzene, p-xylene and styrene, and threonine and serine were the metabolic substrates of 2, 5-dimethyl-pyrazine, 2, 5-dimethyl-3-(3-methylbutyl)-pyrazine, and 3-ethyl-2,5-dimethyl-pyrazine. Therefore, amino acid quantification was conducted to explore the effects on fermentation aroma.

**Figure 3:**
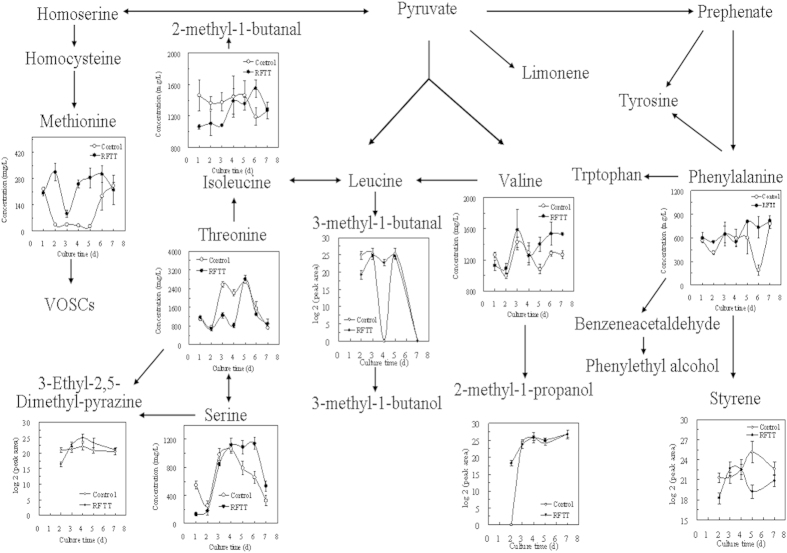
Changes in key VOCs and related amino acids of fermentation sample with RFTT. The key volatile organic compounds were screened by PCA and the Kruskal-Wallis test. The amino acids were detected dynamically and aroma attributes were obtained by sensory evaluation.

A total of 16 amino acids were detected in the fermentation broth and mycelia on Days 2, 3, 4, 5 and 7, including glycine (Gly), alanine (Ala), glutamic acid (Glu), lysine (Lys), arginine (Arg), proline (Pro), valine (Val), tyrosine (Tyr), isoleucine (Ile), tryptophan (Try), serine (Ser), histidine (His), threonine (Thr), methionine (Met), cysteine (Cys) and phenylalanine (Phe). RFTT had different effects on these amino acids. The concentrations of Glu, Ser, His, Arg, Val, Met, Ile and Lys were significantly increased, while the concentrations of Thr and Tyr decreased during the fermentation process. For example, as shown in Fig. 3, the methionine content in the freeze-thaw fermentation sample was higher than in the control, and methionine was the metabolic precursor of volatile organic sulfur-containing compounds (VOSCs), which contributed to the odor descriptor of sulfurous in the fermentation aroma. The 2-methyl-1-butanal, which was important to the formation of the earthy odor component, was produced by leucine and isoleucine. During the fermentation process, the isoleucine content was lower than that in the control from Days 1 to 5 and then became higher. This change might be the reason the odor descriptor of earthy could be identified at the end of fermentation. The decreasing content of 3-methyl-1-butanol resulted in the sensory descriptors of green and malty. The content of threonine (Day 2) with RFTT was lower than that in the control, resulting in decreased content of 3-ethyl-2,5-dimethyl-pyrazine, which could enrich the aroma attribute with nutty and roasted smells. Valine could produce 2-methyl-1-propanal and 2-methyl-1-propanol, and phenylalanine is the metabolic precursor of styrene, benzeneacetaldehyde and phenylethyl alcohol. With the increasing content of these VOCs, the fermentation aroma developed more fruity and floral odors. In conclusion, repeated freeze-thaw treatment mainly caused metabolic changes in 9 key VOCs (1-octen-3-ol, 3-octanol, hexadecanoic acid methyl ester, ethylbenzene, p-xylene, styrene, 3-ethyl-2,5-dimethyl-pyrazine, limonene, and 2,5-dimethyl-3-(3-methylbutyl)-pyrazine) associate with 6 amino acids (methionine, valine, serine, phenylalanine, isoleucine, and threonine). The metabolic changes in these amino acids resulted in changes in key VOCs and an improved aroma.

Amino acids can be converted in many different ways by enzymes such as deaminases, decarboxylases, transaminases (aminotransferases), and lyases. Transamination of amino acids results in the formation of α-keto acids that can be converted into aldehydes by decarboxylation, subsequently, into alcohols or carboxylic acids by dehydrogenation^34^. Many of these compounds are major aroma components. For the aroma of truffle, volatile organic sulfur-containing compounds (VOSCs, such as methanethiol (MTL), dimethyl sulfide (DMS), dimethyl disulfide (DMDS), dimethyl trisulfide (DMTS), 3-(methylthio)propanal (methional) and 3-(methylthio)-1-propanol (methionol)) are key contributors to truffle aroma, because of their characteristic notes for the human nose and very low olfactory threshold^6^,^22^,^35^. Thus, the biosynthesis of the VOCs from amino acids in truffle could be divided into two parts: VOSCs derived from methionine and VOCs derived from nonsulfur amino acid catabolism. (1) The metabolism of methionine to the biosynthesis of VOSCs had been outlined as following. First, methionine is converted to 4-methylthio-2-oxobutyric acid (KMBA) via aminotransferase. Then, KMBA is divided between two pathways, i.e., the Ehrlich and Demethiolation pathways. In the Ehrlich pathway, the KMBA is converted into methional by α-ketoacid decarboxylase, and the methional is further oxidized to methionol by alcohol dehydrogenase. Meanwhile, KMBA is also converted to MTL via demethiolase (also known as C-S lyase), and MTL is spontaneously oxidized to DMS, DMDS and DMTS via the pathway known as the Demethiolation pathway. Our previous work indicated that methionol was the major metabolite produced by the Ehrlich pathway and that DMDS was the major metabolite produced by the Demethiolation pathway^22^,^36^. (2) The branched chain hydrocarbons of VOCs (i.e., 2-methylbutanal, 3-methylbutanal, 2-methylpropanal and 2-phenylethanol) could be produced from nonsulfur amino acid (i.e., isoleucine, leucine, valine and phenylalanine) through the Ehrlich pathway. While, both the Ehrlich pathway and Demethiolation pathway operate in truffles are the matter of speculation, and the candidate genes potentially involved have been proposed for *T. melanosporum*^8^. These would be very useful to construct the metabolite biosynthesis pathway for efficiently aroma-producing during the submerged fermentation of *T. melanosporum*^36^.

In this work, repeated freeze-thaw treatment (RFTT) was adopted to improve the quality of *T. melanosporum* fermentation aroma from the “liked slightly” to the “liked moderately” grade, and the aroma attributes of sulfurous, mushroom and earthy could be detected from RFTT samples for the first time. A total of 29 VOCs were identified, and 9 key discriminative VOCs were screened. Compared to the control without RFTT, the relative abundances of 1-octen-3-ol, 3-octanol, 3-ethyl-2, 5-dimethyl-pyrazine and 2, 5-dimethyl-3-(3-methylbutyl)-pyridine in RFTT samples were higher, while the relative abundances of hexadecanoic acid, methyl ester and limonene were lower, and the relative abundances of ethylbenzene, p-xylene and styrene changed irregularly. A total of 16 amino acids were detected in the fermentation sample. Combined with the aroma attributes, these results suggest that methionine, valine, serine, phenylalanine, isoleucine and threonine may be the potential substrates associated with the metabolism of key discriminative VOCs, ultimately affecting the fermentation aroma. This study indicated that the amino acid metabolism played an important role in the formation of the *T. melanosporum* fermentation aroma.

## Methods

### Chemicals and reagents

2-Methyl-1-propanol, 3-methyl-1-butanol, 3-methyl-butanal, 3-octanone, p-xylene, styrene, benzeneacetaldehyde, phenylethyl alcohol, 4-ethyl-phenol, 2,5-dimethyl-pyrazine, trimethyl-pyrazine, and 3-ethyl-2,5-dimethyl-pyrazine were purchased from Sigma-Aldrich China Inc. (Beijing, China). The 18 amino acid standards, including glycine (Gly), asparagine (Asn), alanine (Ala), glutamic acid (Glu), lysine (Lys), arginine (Arg), proline (Pro), valine (Val), tyrosine (Tyr), isoleucine (Ile), leucine (Leu), tryptophan (Try), serine (Ser), histidine (His), threonine (Thr), methionine (Met), cysteine (Cys) and phenylalanine (Phe), were all purchased from Biosharp.

High-performance liquid chromatography (HPLC)-grade acetonitrile was supplied by Tedia Company (Fairfield, OH, USA). Phenylisothiocyanate (PITC, lot code: 10113227) was purchased from Alfa Aesar Chemical Co. Ltd (Tianjin, China), with a purity higher than 97%. Triethanolamine (TEA, analytical grade) and n-hexane (analytical grade) were purchased from Sinopharm Chemical Reagent Co. Ltd. (Shanghai, China). Pure water was obtained using a Milli-Q purification system (Millipore, Bedford, MA, USA).

### Microorganisms and culture conditions

The strain of *T. melanosporum* was kindly provided by the Mianyang Institute of Edible Fungi (Building No.110, Hongxing Street, Mianyang City, Sichuan Province, and China). The details of the basic culture medium and procedure have been described elsewhere^37^. During the fermentation process, five cultivation time points (Days 2, 3, 4, 5 and 7) were selected to conduct the following treatment. For sampling, five flasks were taken each time.

### Repeated freeze-thaw treatment and sensory evaluation

The fermentation sample (50 mL) was placed in a 250 mL flask and sealed with a rubber stopper. It was then frozen at –20 °C for 55 min then thawed at 30 °C for 5 min in a water bath. The sensory properties of the samples were characterized after 1, 3, 5, and 10 freeze-thaw cycles^23^.

In sensory evaluation, for sampling, five samples were taken each time, and three panelists were asked to describe their degree of liking of the color and overall acceptability by assigning a liking score on a nine-point hedonic scale (1 = disliked extremely; 2 = disliked very much; 3 = disliked moderately; 4 = disliked slightly; 5 = neither liked nor disliked; 6 = liked slightly; 7 = liked moderately; 8 = liked very much; 9 = liked extremely)^38^. The results were tested by one-way analysis of variance (p < 0.05). The aroma attributes of each sample were also recorded.

### Volatile compounds analysis

#### Head space-solid phase microextraction (HS-SPME) capture conditions

The extraction of volatile organic compounds was performed essentially as described by Liu *et al.*^39^. A 75-μm carboxen–polydimethylsiloxane (CAR–PDMS) fiber was used for all analyses. A 5-mL volume of *T. melanosporum* culture (including mycelia and broth) was directly added into a 15-mL vial sealed with one layer of Parafilm-MTM (American National Can, Menasha, WI 54952, USA) and a seal ring, and the nut was tightened to avoid the loss of volatile compounds due to the permeability to gases of this plastic. Afterward, a solid-phase microextraction (SPME) device was manually introduced into the vial and was exposed in the headspace of the vial to extract the VOCs for 30 min at 50 °C. After extraction, the fiber was pulled into the needle assembly, and the SPME device was removed from the vial. The device was then inserted into the injection port of the gas chromatograph (GC) for thermal desorption of the analysts at 270 °C for 2 min.

#### Gas chromatography–mass spectrometry (GC–MS) conditions

VOCs trapped on the fiber were analyzed by GC–MS using an Agilent 7890A GC system equipped with a 5975C quadrupole mass spectrometer (MS) detector (CA, USA). Separations were conducted on an HP-5 ms capillary column (30 m × 0.32 mm i.d., 0.25 μm film thickness) from Agilent Technologies, Inc. (CA, USA). Oven temperature conditions were 40 °C (hold for 1 min), rising to 100 °C at the rate of 10 °C min^−1^ (hold for 3 min), then to 130 °C at 5 °C min^−1^ (hold for 1 min), then to 150 °C at 3 °C min^−1^ (hold for 1 min), and finally to 250 °C at 15 °C min^−1^. Nitrogen (99.999%) was used as the carrier gas at a constant flow rate of 3.0 mL min^−1^. The detector temperature was set at 280 °C. Mass/z detection was obtained using an Agilent mass spectrometer operating in the EI mode (ionization energy, 70 eV, source temperature 200 °C). Data acquisition was performed in scanning mode (mass range m/z 30–500, seven scans per second). Chromatograms and spectra were recorded and processed using Enhanced ChemStation software for GC–MS (Agilent).

#### Identification and relative quantification of compounds

Compound identification was based first on the comparison between the MS for each putative compound and the records in the NIST 2011 Mass Spectral library for a selected number of the compounds by the consistency of GC retention time and mass spectra information generated using analytical grade commercial compounds (Sigma-Aldrich, Spain). A mixture of five standard compounds was injected at regular intervals interspersed throughout the injection series (every 6 samples) and was used as a reference for correction of the instrument variability and fiber aging.

### Amino acid analysis

#### Sample preparation

Each of the 18 amino acids was accurately weighed (Asn 50.2 mg, Glu 50.7 mg, Try 9.7 mg, Ser 10.4 mg, Gly 10.4 mg, His 49.7 mg, Arg 50.5 mg, Thr 25.7 mg, Ala 10.0 mg, Pro 10.2 mg, Tyr 50.5 mg, Val 4.9 mg, Met 10.4 mg, Cys 10.0 mg, Ile 4.9 mg, Leu 5.1 mg, Phe 5.0 mg, and Lys 5.0 mg). The stock solution was prepared by dissolving the above 18 standards in a 100-mL volumetric flask with 0.1 mol/L hydrochloric acid and was then stored in a refrigerator at 4 °C until use. Norleucine was used as the internal standard during the qualification and quantification of amino acids.

During the cultivation time, the truffle fermentation broth (10 mL) and fresh mycelia (1.00 mg) were harvested. First, 10 mL of pure water was added into the fresh mycelia, and ultrasonic disruption was employed to break up the cells. The fermentation broth samples (1 mL) and mycelia samples (1 mL) were centrifuged at 13,400 × *g* for 10 min. A 200-μL aliquot of the supernatant was added into a 1.5-mL Eppendorf tube. Protein precipitation was conducted by adding 400 μL of methanol-acetonitrile solution (v/v, 1:1). The Eppendorf tubes were vortex-mixed and allowed to stand overnight, then were centrifuged at 13,400 × *g* for 10 min. Next, 200 μL of supernatant was transferred into another tube, and 50 μL of norleucine solution (10 mg/L) was added, followed by the addition of 100 μL of phenylisothiocyanate (PITC), acetonitrile solution (0.1 mol/L), 100 μL of triethylamine (TEA), and acetonitrile solution (1 mol/L). The mixture was then shaken and left to stand at room temperature for 1 h. Afterwards, 400 μL of n-hexane was added, and the mixture was shaken thoroughly and then left to stand at room temperature for 20 min. The underlayer of solution was filtered through a 0.45-μm membrane filter for injection^40^,^41^.

#### HPLC conditions

Analyses were primarily performed on an UltiMate3000 liquid chromatographic system with a Diamond C18 column (4.6 mm × 250 mm, 5 μm). The flow rate was 1.0 mL/min, and the sample injection volume was 20 μL. The detection wavelength was set at 254 nm. The column temperature was maintained at 40 °C. The mobile phase consisted of 0.1 mol/L acetate (pH was adjusted to 6.5 with glacial acetic acid)-acetonitrile (93:7) (A) and acetonitrile-water (4:1) (B). The gradient program was as follows: 0–2 min, 0% B, 2–5 min, 0% B, 15–25 min, 10–30% B, 25–33 min, 30–32% B, 33–41 min, 32–45% B, 41–41.1 min, 100% B, 41.1–44 min, 100% B, 44–44.1 min, 100–0% B, and 44.1–50 min, 0% B. The chromatographic profiles of the blank control solution, the mixed amino acid standard solution, and the fermentation sample on Day 5 of cultivation time are shown in Supplementary Fig. S1. The blank control solution was obtained by preparing 0.1 mol/L hydrochloric acid according to the sample processing procedure.

The method was validated by analyzing calibration standards in triplicate for each unlabeled compound to obtain the retention time, standard curve, R^2^, lower limit of quantitation (LLOQ) and higher limit of quantitation (HLOQ) (see Supplementary Table S1).

### Statistical analysis

For sampling, five flasks were taken each time. For analyses of variance (ANOVA), a multiple comparison between mean values was performed using the least significant difference (LSD) test (P ≤ 0.05) with SPSS 19.0 software (SPSS Inc., 233 South Wacker Drive, 11th Floor, Chicago, USA). When PCA was performed, the data were first log 2-transformed (log 2 (peak area)). Simca-P 11.5 software was used (Umetrics, Umea, Sweden, http://www.umetrics.com/simca). Significant differences in metabolites between treatment and control or among genotypes were tested using the Kruskal-Wallis test (P ≤ 0.05) on SPSS version 19.0 for Windows (SPSS Inc., 233 South Wacker Drive, 11th Floor, Chicago, USA). Metabolic pathways were constructed based on research studies and the KEGG metabolic database.

## Additional Information

**How to cite this article**: Xiao, D.-R. *et al.* Aroma improvement by repeated freeze-thaw treatment during *Tuber melanosporum* fermentation. *Sci. Rep.* **5**, 17120; doi: 10.1038/srep17120 (2015).

## Supplementary Material

Supplementary Information

## Figures and Tables

**Table 1:** Effects of repeated freeze-thaw treatment on the aroma attribute(s) in the submerged fermentation of *T. melanosporum.*

	Control^a^	1 cycle^b^	3 cycles	5 cycles	10 cycles
Day 1	medium, alcohol, sweet	medium, sweet	medium, sweet, **earthy**	medium, sweet, **earthy**	medium, sweet, **mushroom**
Day 2	medium, alcohol, flowery, sweet	medium, sweet	alcohol, flowery, sweet, **mushroom**	medium, alcohol, flowery, sweet, **mushroom**	medium, alcohol, green, sweet, **mushroom**
Day 3	alcohol, fruity, sweet	alcohol, flowery, fruity, sweet, **sulfurous**	alcohol, flowery, sweet, **sulfurous**	alcohol, flowery, fruity, green, sweet, **sulfurous**	alcohol, flowery, fruity, sweet, sulfurous, **mushroom**
Day 4	alcohol, flowery, fruity, sweet	alcohol, flowery, green, sweet	alcohol, fruity, sweet, **earthy**	alcohol, flowery, fruity, sweet, **sulfurous**	alcohol, flowery, fruity, sweet, **sulfurous**
Day 5	alcohol, flowery, fruity, green, sweet	alcohol, flowery, sweet	alcohol, flowery, fruity, green, sweet	alcohol, flowery, fruity, sweet	alcohol, flowery, fruity, sweet
Day 6	alcohol, flowery, fruity, sweet, hay	alcohol, flowery, fruity, green, sweet, **sulfurous**	alcohol, flowery, fruity, green, sweet, mold	alcohol, flowery, green, sweet, mold	alcohol, flowery, fruity, sweet, mold
Day 7	alcohol, flowery, fruity, sweet, earthy, mold	alcohol, flowery, sweet, mold	alcohol, fruity, sweet, hay, mold	alcohol, flowery, green, sweet, mold	alcohol, flowery, green, mold
Day 8	sweet, mold	flowery, sweet, hay, mold	alcohol, sweet, mold	sweet, mold	alcohol, **earthy, mold**

^a^Fermentation sample without RFTT.

^b^Fermentation sample frozen at −20 °C for 55 min, then thawed at 30 °C for 5 min with 1 cycle.

**Table 2:** Volatile organic compounds (VOCs) detected via SPME-GC–MS of *T. melanosporum* fermentation.

NO.^a^	LRI^b^	Compound^c^	Detected mass m/z^d^	Log_2_(peak area)^e^									
				Day 2		Day 3		Day 4		Day 5		Day 7	
				Control^f^	RFTT^g^	Control	RFTT	Control	RFTT	Control	RFTT	Control	RFTT
		Alcohols											
1	<600	Ethanol**	31.1	24.93 ± 0.61	18.26 ± 1.09	26.98 ± 1.10	25.43 ± 1.79	27.38 ± 0.70	27.25 ± 1.29	27.62 ± 0.76	27.16 ± 1.11	27.31 ± 1.62	29.07 ± 1.32
2	623	2-Methyl-1-propanol**	43.1	—	18.23 ± 0.93	24.30 ± 0.71	23.63 ± 1.07	25.61 ± 0.80	25.72 ± 1.43	24.15 ± 0.78	25.10 ± 0.51	26.70 ± 1.29	26.78 ± 1.06
3	730	3-Methyl-1-butanol*	55.1	26.98 ± 0.93	23.07 ± 1.83	27.70 ± 2.00	26.81 ± 1.76	29.29 ± 1.47	27.60 ± 1.73	26.20 ± 2.23	27.99 ± 1.13	28.28 ± 1.41	27.41 ± 1.23
4	866	(E)-4-Hexen-1-ol**	67.1	—	—	21.71 ± 1.08	—	—	—	—	22.95 ±1.46	—	—
5	973	1-Octen-3-ol**	57.1	21.99 ± 1.07	20.37 ± 0.81	21.41 ± 1.08	24.52 ± 0.92	21.93 ± 1.25	24.35 ± 0.65	21.49 ± 0.79	23.39 ± 0.75	—	—
6	989	3-Octanol**	59.1	—	19.21 ± 1.19	—	—	—	21.58 ± 0.88	—	23.48 ± 0.64	—	—
7	1555	[S-(Z)]-3,7,11- Trimethyl-1,6,10- dodecatrien-3-ol**	69.1	21.04 ± 0.96	—	—	—	—	—	—	—	—	—
		Aldehyde											
8	654	3-Methyl-butanal**	41.1	24.92 ± 1.18	19.20 ± 1.30	25.33 ± 1.60	24.82 ± 0.46	—	22.71 ± 0.90	25.38 ± 1.49	24.53 ± ± 0.96	—	—
		Ketone											
9	982	3-Octanone	57.1	21.13 ± 0.72	16.18 ± 0.58	—	—	19.74 ± 0.89	21.32 ± 0.80	20.51 ± 0.66	21.16 ± 0.54	19.74 ± 1.43	24.52 ± 1.78
		Esters and acid											
10	1239	Benzeneacetic acid, ethyl ester**	91.1	—	—	—	—	19.86 ± 1.02	19.56 ± 1.46	20.08 ± 0.59	—	—	—
11	1754	Tetradecanoic acid**	73.1	—	12.11 ± 0.72	—	—	—	—	—	—	—	—
12	1914	Hexadecanoic acid, methyl ester**	74	20.26 ± 0.74	—	—	—	—	18.74 ± 0.57	18.74 ± 0.43	—	—	—
13	1950	n-Hexadecanoic acid**	73	18.43 ± 0.93	—	—	—	—	—	—	—	—	—
		Aromaticcompounds											
14	859	Ethylbenzene**	91	—	15.29 ± 0.81	21.13 ± 0.72	19.72 ± 0.71	21.36 ± 0.78	20.97 ± 0.54	21.18 ± 0.54	—	—	—
15	865	p-Xylene**	91.1	21.21 ± 0.86	15.69 ± 0.72	21.41 ± 0.84	19.85 ± 0.87	22.36 ± 0.70	22.10 ± 1.36	23.18 ± 0.30	—	—	19.99 ± 1.12
16	887	Styrene**	104.1	21.28 ± 0.72	18.27 ± 0.81	21.33 ± 0.82	22.66 ± 0.99	22.54 ± 0.83	22.53 ± 1.57	25.14 ± 1.62	19.24 ± 1.00	22.67 ± 1.03	20.89 ± 0.87
17	1036	Benzeneaceta-ldehyde**	91.1	20.53 ± 0.71	—	18.70 ± 0.70	18.66 ± 0.66	18.77 ± 0.76	18.87 ± 0.83	20.74 ± 1.14	21.07 ± 0.71	19.33 ± 1.27	18.42 ± 0.98
18	1112	Phenylethyl alcohol*	91.1	27.14 ± 1.87	21.00 ± 1.60	28.87 ± 1.09	31.28 ± 2.13	28.85 ± 1.64	29.97 ± 1.51	29.43 ± 1.76	28.90 ± 1.29	29.16 ± 1.83	30.46 ± 2.76
19	1159	4-Ethyl-phenol*	107.1	—	—	—	—	—	—	19.46 ± 1.47	18.31 ± 1.05	—	—
20	1219	Benzothiazole**	135	17.58 ± 0.79	—	—	—	—	—	—	18.17 ± 1.06	—	—
		Nitrogen											
21	<600	2-Hydroxy-propanamide**	45.1	—	15.02 ± 1.23	—	—	—	—	—	22.28 ± 0.78	—	—
22	906	2,5-Dimethyl-pyrazine**	108.1	22.19 ± 0.92	18.09 ± 1.04	23.83 ± 1.19	24.92 ± 0.98	24.21 ± 1.30	23.07 ± 0.99	22.96 ± 0.63	—	25.62 ± 1.38	24.10 ± 0.45
23	997	Trimethyl-pyrazine**	42.1	22.98 ± 1.23	20.20 ± 1.30	21.60 ± 1.35	24.36 ± 0.68	21.26 ± 0.82	—	—	—	25.38 ± 1.38	23.75 ± 0.87
24	1069	3-Ethyl-2,5-dimethyl- pyrazine**	135.1	20.92 ± 0.78	16.45 ± 0.74	21.20 ± 0.96	22.69 ± 0.79	22.15 ± 1.05	—	20.92 ± 0.90	23.30 ± 1.43	20.48 ± 1.06	21.16 ± 0.52
25	1308	2,5-Dimethyl-3- (3-methylbutyl)- pyrazine**	122.1	18.55 ± 1.08	14.61 ± 1.02	18.31 ± 0.83	20.72 ± 0.97	19.27 ± 1.09	21.28 ± 0.91	—	—	18.39 ± 0.85	21.30 ± 1.79
		Sulfur											
26	976	3-(Methylthio)-1- propanol**	106.1	22.70 ± 1.01	—	—	—	—	—	—	—	—	—
		Alkene											
27	1021	Limonene**	68.1	19.67 ± 0.82	—	19.12 ± 0.91	—	—	—	19.58 ± 0.80	—	—	—
28	1310	2,3,4-Trimethyl-1,4- pentadiene**	95.1	—	—	—	—	—	23.19 ± 1.87	25.31 ± 0.79	26.67 ± 1.71	21.56 ± 0.93	—
29	1449	(E)-7,11-Dimethyl-3- methylene-1,6,10- dodecatriene**	41.1	20.31 ± 1.02	—	—	—	—	—	—	—	—	—

^a^Compound number (No.) according to linear retention index (LRI) from low to high.

^b^LRI: Linear retention index calculated on an HP-5 column.

^c^Compounds with * exhibited a significant difference, p < 0.05, compounds with **exhibited a highly significant difference, p < 0.001.

^d^Specific ion (m/z) selected for relative quantification of each compound.

^e^Peak area of each compound, with log2-transformed value, calculated as the average of five replicates (n = 5).

^f^Control: fermentation sample without repeated freeze-thaw treatment.

^g^RFTT: fermentation sample with repeated freeze-thaw treatment.
