# Supplementary material for: Aroma improvement by repeated freeze-thaw treatment during Tuber melanosporum fermentation
Source: Sci Rep. 2015 Nov 26;5:17120. doi: 10.1038/srep17120 (PMC4660818; doi:10.1038/srep17120)
Supplement: Supplementary Information [file srep17120-s1.doc]

**Aroma improvement by repeated freeze-thaw treatment during *Tuber melanosporum* fermentation**

Deng-Rong Xiao ^a, 1^, Rui-Sang Liu ^a, 1^, Long He ^a, 1^, Hong-Mei Li ^a^, Ya-Ling Tang ^b^,

Xin-Hua Liang ^b^, Tao Chen ^c^, Ya-Jie Tang ^a^,*

^a^ Key Laboratory of Fermentation Engineering (Ministry of Education), Hubei Provincial Cooperative Innovation Center of Industrial Fermentation, Hubei University of Technology, Wuhan 430068 China

^b^ State Key Laboratory of Oral Diseases, West China Hospital of Stomatology, Sichuan University, Chengdu 610041 China

^c^ Key Laboratory of Systems Bioengineering (Ministry of Education), School of Chemical Engineering and Technology, Tianjin University, Tianjin 300072 China

^1^ These authors contributed equally to this work.

*Corresponding author. Tel. & Fax: +86-27-59750491. E-mail: yajietang@hotmail.com

**Supplementary information**

Supplementary Table S1 Standard curve of 18 amino acids.

Supplementary Table S2 The contribution of metabolites of *T. melanosporum* fermentation with repeated freeze-thawed treatment (RFTT) to the first, second, third and fourth principal components.

Supplementary Table S3 Difference analysis of VOCs profiles produced from *T. melanopsorum* fermentation sample treated by RFTT.

Supplementary Figure [S1] Representative HPLC chromatograms of amino acids obtained from (A) the blank control solution, (B) the mixed standards solution of amino acids, (C) Fermentation broth and mycelia of Day 5 with RFTT and without RFTT.

Supplementary Figure [S2] Component of volatile organic compounds from T. melanosporum fermentation (A) Day 2-control, (B) Day 2-RFTT, (C) Day 3-control, (D) Day 3-RFTT, (E) Day 4-control, (F) Day 4-RFTT, (G) Day 5-control, (H) Day 5-RFTT, (I) Day 7-control, and (J) Day 7-RFTT.

**Table S1 Standard curve** of 18 amino acids (AAs).

| AAs | Retention time (min) | Standard curve | R^2^ | Linear dynamic range(LLOQ ^a^-HLOQ ^b^) (mg/L-mg/L) |
| --- | --- | --- | --- | --- |
| Glu | 9.680 | y = 0.2202x + 1.5227 | 0.9950 | 2.84-19.90 |
| Asn ^c^ | 9.543 | y = 0.4392x - 0.8013 | 0.9955 | 2.21-19.85 |
| Ser | 12.713 | y = 1.2662x - 3.764 | 0.9982 | 2.03-40.59 |
| Gly | 13.220 | y = 1.8914x + 0.2177 | 0.9996 | 2.04-40.80 |
| His | 14.140 | y = 0.3023x + 0.2856 | 0.9986 | 2.26-40.70 |
| Arg | 14.787 | y = 0.8875x -9.0646 | 0.9979 | 2.24-10.10 |
| Thr | 16.907 | y = 0.7374x - 12.198 | 0.9974 | 2.26-20.35 |
| Ala | 18.067 | y = 1.4812x - 1.0589 | 0.9987 | 2.02-40.30 |
| Pro | 20.143 | y = 1.2933x + 1.394 | 0.9985 | 2.04-40.8 |
| Tyr | 26.273 | y = 0.8245x + 1.0004 | 0.9978 | 2.03-40.60 |
| Val | 27.130 | y = 1.0773x + 3.1334 | 0.9975 | 2.26-40.60 |
| Met | 28.063 | y = 0.9049x - 0.7705 | 0.9987 | 2.02-40.4 |
| Cys | 28.637 | y = 0.416x - 25.581 | 0.9998 | 2.03-5.08 |
| Ile | 30.300 | y = 1.0652x + 1.064 | 0.9990 | 2.02-40.30 |
| Leu ^c^ | 31.183 | y = 1.1039x + 3.0617 | 0.9988 | 1.98-9.88 |
| Phe | 32.860 | y = 0.9164x - 2.3485 | 0.9978 | 3.38-40.60 |
| Trp | 34.037 | y = 0.8661x + 0.205 | 0.9996 | 2.00-40.00 |
| Lys | 36.733 | y = 1.0278x - 1.3603 | 0.9991 | 2.26-40.70 |

^a^ Lower limit of quantitation

^b^ Higher limit of quantitation

^c^ Not detected in this study.

**Table S2 The contribution of metabolites of *T. melanosporum* fermentation with repeated freeze-thaw treatment (RFTT) to the first, second, third and fourth principal components**.

| NO. ^a^ | Compounds | PC1 (23.2%) ^b^ | PC2 (20.8%) | PC3 (17.8%) | PC4 (13.7%) |
| --- | --- | --- | --- | --- | --- |
| 1 | Ethanol | 0.0683 | 0.1440 | -0.3091 | 0.2170 |
| 2 | 2-Methyl-1-propanol | 0.3671 | 0.0906 | -0.0494 | 0.0532 |
| 3 | 3-Methyl-1-butanol | 0.0304 | 0.1110 | -0.1952 | 0.2197 |
| 4 | (E)-4-Hexen-1-ol | 0.0808 | -0.2095 | -0.0660 | 0.1272 |
| 5 | 1-Octen-3-ol | -0.0564 | -0.1428 | -0.0908 | -0.3328 |
| 6 | 3-Octanol | 0.1245 | -0.2999 | 0.0446 | -0.1410 |
| 7 | [S-(Z)]-3,7,11-Trimethyl-1,6,10-dodecatrien-3-ol | -0.3781 | -0.0459 | -0.0341 | 0.0336 |
| 8 | 3-Methyl-butanal | -0.0971 | -0.1963 | -0.1099 | -0.2676 |
| 9 | 3-Octanone | -0.0260 | -0.0946 | -0.1056 | 0.0937 |
| 10 | Benzeneacetic acid, ethyl ester | 0.0907 | 0.1008 | -0.2417 | -0.2760 |
| 11 | Tetradecanoic acid | 0.0262 | -0.1418 | 0.3414 | -0.2025 |
| 12 | Hexadecanoic acid, methyl ester | -0.1997 | -0.0165 | -0.2563 | -0.2463 |
| 13 | n-Hexadecanoic acid | -0.3782 | -0.0459 | -0.0341 | 0.0335 |
| 14 | Ethylbenzene | 0.1175 | 0.1345 | -0.0320 | -0.3976 |
| 15 | p-Xylene | -0.1160 | 0.2091 | -0.0368 | -0.3172 |
| 16 | Styrene | 0.0121 | 0.2264 | -0.2763 | -0.0877 |
| 17 | Benzeneacetaldehyde | -0.0427 | 0.0910 | -0.3657 | 0.2009 |
| 18 | Phenylethyl Alcohol | 0.0953 | 0.1635 | -0.2087 | 0.1141 |
| 19 | 4-Ethyl-phenol | 0.0975 | -0.2412 | -0.2662 | -0.0045 |
| 20 | Benzothiazole | -0.2049 | -0.2851 | -0.1050 | 0.1679 |
| 21 | 2-Hydroxy-propanamide | 0.1005 | -0.3717 | 0.1087 | 0.0455 |
| 22 | 2,5-Dimethyl-pyrazine | -0.0726 | 0.3743 | 0.0419 | -0.1047 |
| 23 | Trimethyl-pyrazine | -0.1248 | 0.2143 | 0.2854 | 0.1905 |
| 24 | 3-Ethyl-2,5-dimethyl-pyrazine | -0.0489 | -0.0160 | 0.0385 | 0.2856 |
| 25 | 2,5-Dimethyl-3-(3-methylbutyl)-pyrazine | -0.0854 | 0.2823 | 0.1958 | 0.0177 |
| 26 | 3-(Methylthio)-1-propanol | -0.3781 | -0.0455 | -0.0345 | 0.0340 |
| 27 | Limonene | -0.2221 | 0.0060 | -0.1702 | -0.1142 |
| 28 | 2,3,4-Trimethyl-1,4-pentadiene | 0.1537 | -0.1625 | -0.2814 | 0.0072 |
| 29 | (E)-7,11-Dimethyl-3-methylene-1,6,10-Dodecatriene | -0.3784 | -0.0465 | -0.0335 | 0.0331 |

^a^ Compound number (NO.) according to linear retention index (LRI) from low to high.

^b^ The percentage of the total data that the principal component 1 could explain.

**Table S3 Difference analysis of volatile organic compounds (VOCs) profiles produced from *T. melanopsorum* fermentation sample treated by RFTT.**

| NO. ^a^ | Compound | RFTT of each time-point ^b^ | | | | |
| --- | --- | --- | --- | --- | --- | --- |
|  |  | Day 2 | Day 3 | Day 4 | Day 5 | Day 7 |
| 1 | Ethanol | Y |  |  |  |  |
| 2 | 2-Methyl-1-propanol | Y |  |  |  |  |
| 3 | 3-Methyl-1-butanol |  |  |  |  |  |
| 4 | (E)-4-Hexen-1-ol |  | Y |  | Y |  |
| 5 | 1-Octen-3-ol |  | Y | Y | Y |  |
| 6 | 3-Octanol | Y |  | Y | Y |  |
| 7 | [S-(Z)]-3,7,11-Trimethyl-1,6,10-dodecatrien-3-ol | Y |  |  |  |  |
| 8 | 3-Methyl-butanal |  |  | Y |  |  |
| 9 | 3-Octanone | Y |  | Y |  | Y |
| 10 | Benzeneacetic acid, ethyl ester |  |  |  | Y |  |
| 11 | Tetradecanoic acid | Y |  |  |  |  |
| 12 | Hexadecanoic acid, methyl ester | Y |  | Y | Y |  |
| 13 | n-Hexadecanoic acid | Y |  |  |  |  |
| 14 | Ethylbenzene | Y | Y |  | Y |  |
| 15 | p-Xylene | Y | Y |  | Y | Y |
| 16 | Styrene |  | Y |  | Y | Y |
| 17 | Benzeneacetaldehyde | Y |  |  |  |  |
| 18 | Phenylethyl Alcohol |  |  |  |  |  |
| 19 | 4-Ethyl-phenol |  |  |  |  |  |
| 20 | Benzothiazole | Y |  |  | Y |  |
| 21 | 2-Hydroxy-propanamide | Y |  |  | Y |  |
| 22 | 2,5-Dimethyl-pyrazine |  |  |  | Y |  |
| 23 | Trimethyl-pyrazine |  | Y | Y |  |  |
| 24 | 3-Ethyl-2,5-dimethyl-pyrazine | Y | Y | Y | Y |  |
| 25 | 2,5-Dimethyl-3-(3-methylbutyl)-pyrazine |  | Y | Y |  | Y |
| 26 | 3-(Methylthio)-1-propanol | Y |  |  |  |  |
| 27 | Limonene | Y | Y |  | Y |  |
| 28 | 2,3,4-Trimethyl-1,4-pentadiene |  |  | Y |  | Y |
| 29 | (E)-7,11-Dimethyl-3-methylene-1,6,10-Dodecatriene | Y |  |  |  |  |

^a^ Compound number (NO.) according to linear retention index (LRI) from low to high,

^b^ RFTT: Repeated freeze-thaw treatment, fermentation samples frozen at -20 °C for 55 min and then thawed at 30 °C for 5 min for ten cycles.

^c^ “Y”: Compounds to be significant difference by principal component analysis and Kruskal–Wallis test.

^d^ The blank indicated “NS”: compounds to be non-significant difference by principal component analysis and Kruskal–Wallis test.

**
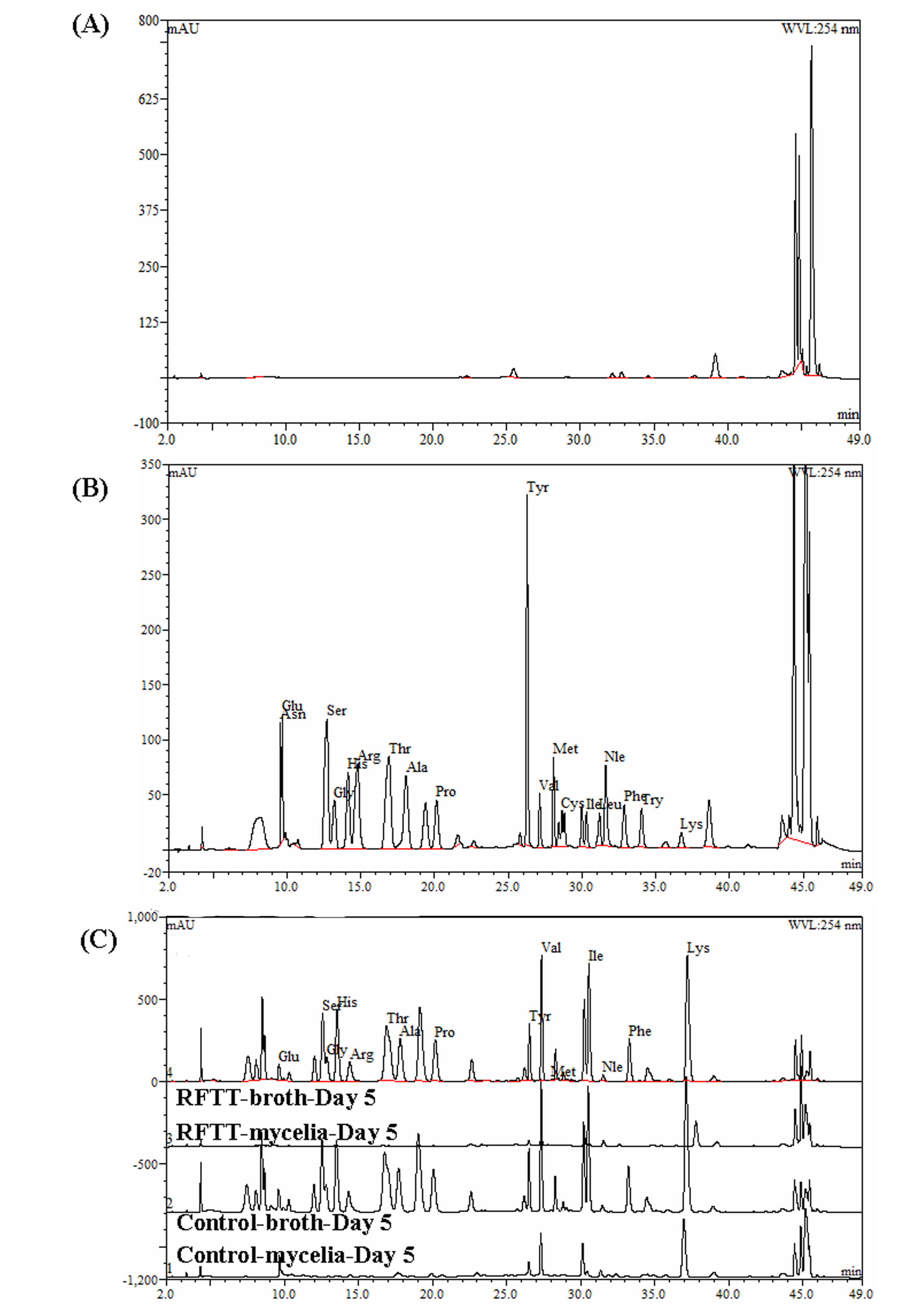
**

Supplementary Figure [S1] Tang et al.

**
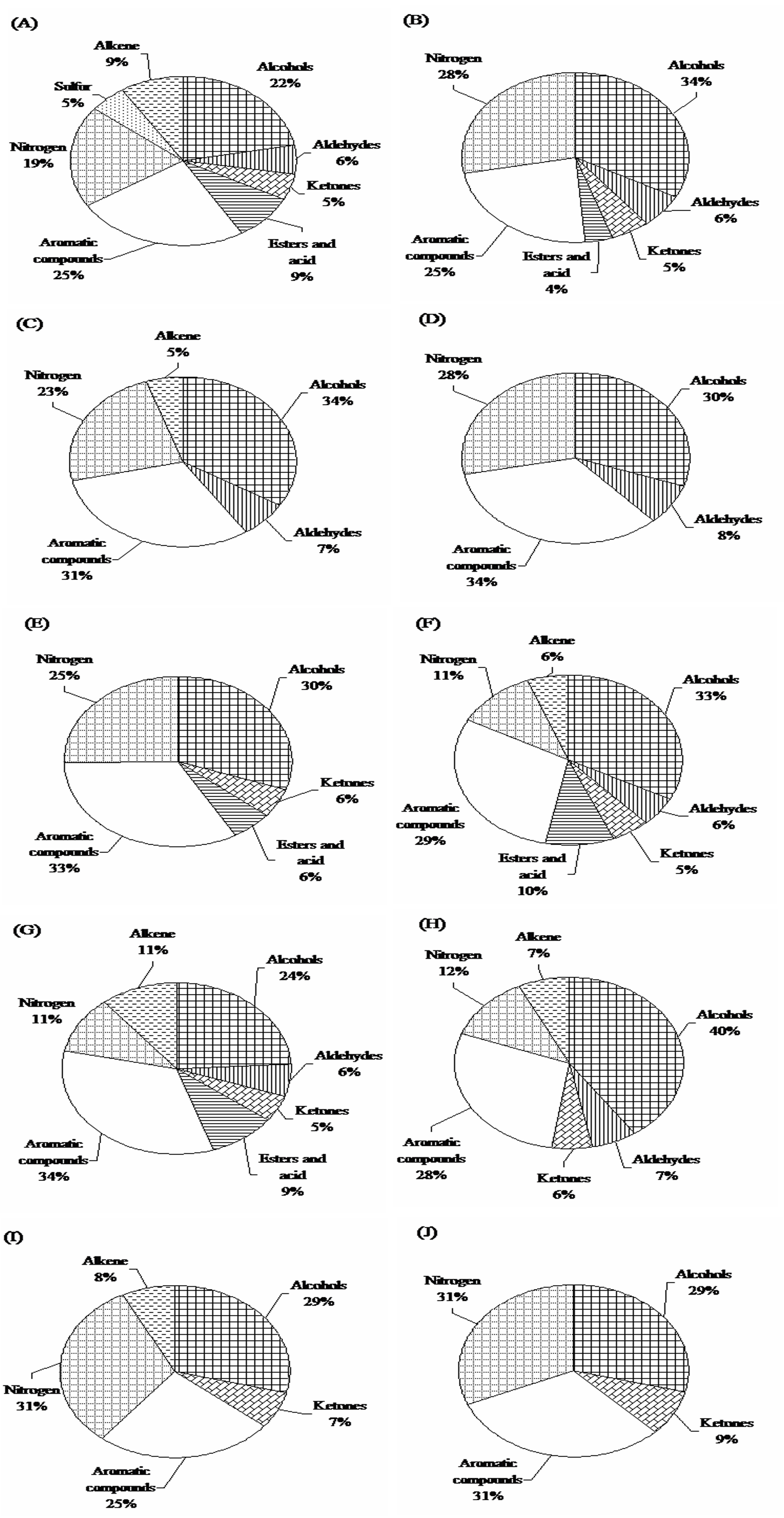
**

Supplementary Figure [S2] Tang et al.
